# The Effect of Introducing Dinoprostone Vaginal Inserts (DVI) in Cases of Term Prelabor Rupture of Membranes With Unfavorable Cervix

**DOI:** 10.7759/cureus.92480

**Published:** 2025-09-16

**Authors:** Ryosuke Shindo, Shigeru Aoki, Sayuri Nakanishi, Soichiro Obata, Etsuko Miyagi

**Affiliations:** 1 Perinatal Center, Yokohama City University Medical Center, Yokohama, JPN; 2 Obstetrics and Gynecology, Yokohama City University, Yokohama, JPN; 3 Oerinatal Center, Yokohama City University Medical Center, Yokohama, JPN; 4 Perinatal Center for Maternity and Neonates, Yokohama City University Medical Center, Yokohama, JPN; 5 Obstetrics and Gynecology, Yokohama City University School of Medicine, Yokohama, JPN

**Keywords:** cervical ripening, chorioamnionitis, dinoprostone, labor induced, premature rupture of fetal membranes

## Abstract

Aim: To evaluate the efficacy and safety of dinoprostone vaginal inserts (DVI) for labor induction in women with term prelabor rupture of membranes (PROM) and an unfavorable cervix.

Methods: This retrospective, single-center observational study included singleton, cephalic pregnancies at ≥37 weeks’ gestation admitted for PROM (August 2017-December 2024). Exclusion criteria were spontaneous labor onset, cesarean delivery without induction, or a Simplified Bishop score (SBS) ≥6. Prior to DVI availability (2017-2020), intravenous oxytocin administration was performed in all cases; after 2020, DVI was administered when SBS ≤5, followed by oxytocin as needed. Sixty matched cases per group were analyzed following propensity score matching for maternal age, height, weight at delivery, and SBS. Primary outcomes included vaginal delivery rate, labor duration, and oxytocin use; secondary outcomes included chorioamnionitis, postpartum hemorrhage, and neonatal morbidity.

Results: Vaginal delivery rates were comparable (DVI 78.3% vs. oxytocin 73.3%, p = 0.52). Median time from PROM to delivery was similar (DVI 47.0 h vs. oxytocin 51.9 h). Oxytocin use was significantly lower in the DVI group (46.7% vs. 100%, p < 0.0001). No significant differences were observed in chorioamnionitis or neonatal outcomes. Among vaginal deliveries, more women in the DVI group delivered within 24 or 48 hours, although this difference was not statistically significant.

Conclusion: In PROM with an unfavorable cervix, DVI use did not increase adverse outcomes and was associated with reduced oxytocin use. While differences in delivery timing were not statistically significant, DVI may provide clinical benefits, supporting the need for further prospective studies.

## Introduction

Prelabor rupture of membranes (PROM) at term occurs in approximately 8% of pregnancies. Following PROM, about 50% of women enter labor within 24 hours, and 95% within 72 hours. However, expectant management increases the risk of intrauterine infection compared to active management through labor induction [1-3]. Therefore, labor induction is generally recommended for term PROM. Nonetheless, there is ongoing debate about whether cervical ripening procedures are necessary in cases of an unfavorable cervix.

Among cervical ripening methods, mechanical approaches have been associated with an increased risk of chorioamnionitis [4] and have not demonstrated clear benefits over oxytocin alone [5]; thus, they are not generally recommended. In contrast, findings regarding the use of vaginal prostaglandin (PG) E2 agents are mixed - some studies report no advantage compared to oxytocin alone [6,7] while others show higher rates of vaginal delivery within 24 hours [8]. Importantly, several reports indicate that these agents do not increase the risk of complications such as chorioamnionitis [9-11], and their administration may be considered prior to initiating oxytocin.

In Japan, approval for intravaginal PG agents occurred later than in many other countries, with the first approval of the dinoprostone vaginal insert (DVI) granted in 2020.

The aim of this study was to evaluate the efficacy of DVI as a method of labor induction in cases of PROM with cervical insufficiency.

## Materials and methods

Study design and participants

This single-center observational study was conducted in accordance with the Declaration of Helsinki and was approved by the Ethics Committee of Yokohama City University (approval number: F221000012). As this was a retrospective observational study, individual informed consent was not obtained from participants. Instead, information about the study was made publicly available on the hospital’s website, and participants were given the opportunity to opt out.

The study included women admitted to Yokohama City University Medical Center between August 2017 and December 2024 due to PROM, who delivered a singleton infant at 37 weeks’ gestation or later, as well as their infants. The facility is a tertiary care hospital located in an urban area of Japan, with approximately 1,000 deliveries per year. At this institution, spontaneous labor is awaited for at least 12 hours in cases of PROM without labor; if labor does not occur, induction is initiated. Prophylactic antibiotics are not routinely administered. Before the introduction of DVI (August 2017 to May 2020), intravenous oxytocin was administered regardless of cervical status. Cervical ripening was assessed using the Simplified Bishop score (SBS), a condensed version of the traditional Bishop score [12]. The SBS evaluates three parameters-cervical dilation, effacement, and fetal head descent - each on a scale of 0 to 3, for a total possible score of 9. It has been reported that an SBS of 6 or higher is as effective, or more effective, than the traditional Bishop score in predicting successful vaginal delivery [13]. In this study, SBS was used as an indicator of cervical ripening. Cases were excluded if they involved non-cephalic presentation, previous uterine surgery, or the use of epidural anesthesia. Additionally, cases in which spontaneous labor began after admission, cesarean delivery was performed without labor induction, or cervical ripening was already favorable (SBS ≥6) at the time of induction were also excluded.

After the introduction of DVI (June 2020 to December 2024), DVI was administered prior to oxytocin when the SBS was ≤5. Prophylactic antibiotics were administered at the time of DVI insertion. Oxytocin was initiated when the SBS reached ≥6.

Instructions for DVI use

DVI was inserted into the vagina by the attending obstetrician. During placement, uterine contraction frequency and fetal heart rate were monitored via cardiotocography (CTG). The insert was removed 12 hours after insertion, unless one of the following conditions occurred earlier: regular uterine contractions every 3 minutes sustained for 30 minutes; tachysystole (more than five contractions in 10 minutes); rupture of membranes (spontaneous or artificial); fetal distress or signs of non-reassuring fetal status (NRFS); or maternal symptoms such as nausea, vomiting, hypotension, or other systemic side effects. Only a single dose of DVI was administered. After removal, intravenous oxytocin was given at the discretion of the attending obstetrician.

Use of oxytocin

For labor induction, oxytocin was diluted in 500 mL of 5% dextrose solution at a concentration of five units and administered at an initial rate of 12 mL/h (2 mU/min). During administration, CTG was used to monitor for frequent uterine contractions and NRFS. The infusion rate was increased by 12 mL/h (2 mU/min) every 30 minutes, up to a maximum of 120 mL/h (20 mU/min).

Background and outcomes

Maternal background variables included maternal age (years), height (cm), pre-pregnancy weight (kg), pre-pregnancy body mass index (BMI, kg/m^2^), parity (primiparous or multiparous), and SBS. Maternal outcomes included vaginal delivery rate, time from PROM to delivery (hours), oxytocin use, clinical chorioamnionitis, intrapartum blood loss (g), and postpartum hemorrhage (PPH). Intrapartum blood loss was defined as blood loss from the onset of the first stage of labor until 2 hours postpartum. Clinical chorioamnionitis was defined according to Lencki’s diagnostic criteria [14]: a maternal temperature of ≥38 °C and one or more of the following: maternal tachycardia (>100 bpm), uterine tenderness, leukocytosis (white blood cell count >15,000/μL), or foul-smelling vaginal discharge. Alternatively, the diagnosis could be made if all four criteria were met in the absence of fever. PPH was defined as intrapartum blood loss of ≥500 g for vaginal deliveries and ≥1,000 g for cesarean deliveries. Neonatal outcomes included birth weight (g), gestational age (weeks), Apgar score at 5 minutes (<7), neonatal intensive care unit (NICU) admission, and neonatal mortality.

For subgroup analysis, cases resulting in vaginal delivery were extracted. In addition to the above variables, the proportion of deliveries occurring within 24 hours and 48 hours from the initiation of labor induction was compared.

Statistical analyses

The chi-square test was used to compare categorical variables, while the Wilcoxon rank-sum test was applied to compare continuous variables. A p-value < 0.05 was considered statistically significant. Propensity score matching was performed at a 1:1 ratio based on maternal age, height, birth weight, and SBS. A caliper width of 0.2 and a random seed of 111 were used. Statistical analyses were conducted using JMP Pro version 15 (JMP Statistical Discovery LLC).

## Results

A total of 1,463 patients were hospitalized for PROM during the study period. Of these, cases were excluded if spontaneous labor occurred after admission, cesarean delivery was performed without induction, or the cervix was deemed favorable (SBS ≥6) at the time of induction. After applying these criteria, 236 cases remained. Patients were categorized into an oxytocin group (n = 165) and a DVI group (n = 71). Following propensity score matching, 60 cases were selected from each group. Maternal background characteristics after matching are presented in Table 1. The median SBS prior to the initiation of labor induction was 2.

**Table 1:** Maternal background characteristics after propensity score matching DVI: dinoprostone vaginal insert; IQR: interquartile range; BMI: body mass index

	Oxytocin Group (N = 60)	DVI Group (N = 60)
Age, years, median (IQR)	32.5 (29.0-37.8)	32.0 (30.0-36.0)
Height, cm, median (IQR)	159 (153-163)	159 (154-163)
Pre-pregnancy weight, kg, median (IQR)	52.0 (48.0-57.0)	53.0 (48.3-60.0)
Pre-pregnancy BMI, kg/m^2^, median (IQR)	20.8 (19.1-23.0)	21.0 (19.9-23.2)
Weight at delivery, kg, median (IQR)	63.2 (57.8-70.7)	64.2 (59.2-70.8)
BMI at delivery, kg/m^2^, median (IQR)	24.8 (23.1-28.3)	25.4 (23.6-27.1)
Nulliparous, n (%)	50 (83.3)	50 (83.3)
Simplified Bishop Score, median (range)	2 (0-5)	2 (0-5)

 Maternal and neonatal outcomes are presented in Table 2. The vaginal delivery rate was 44 (73.3%) in the oxytocin group and 47 (78.3%) in the DVI group, with no statistically significant difference between the groups (p = 0.52). The median time from rupture of membranes to initiation of labor induction was 27.7 hours in the oxytocin group and 27.5 hours in the DVI group, also showing no significant difference. Similarly, the median time from rupture of membranes to delivery was 51.9 hours and 47.0 hours, respectively, with no significant difference. In contrast, the frequency of oxytocin use was significantly lower in the DVI group 28 (46.7%) compared to the oxytocin group 60 (100%) (p < 0.0001). The incidence of clinical chorioamnionitis was 8 (13.3%) in the oxytocin group and 5 (8.3%) in the DVI group, with no significant difference (p = 0.56). There were also no significant differences in intrapartum blood loss or the incidence of postpartum hemorrhage. Neonatal outcomes were likewise comparable between the groups.

**Table 2:** Maternal and neonatal outcomes after propensity score matching DVI: dinoprostone vaginal insert; IQR: interquartile range; PROM: prelabor rupture of membranes; IOL: induction of labor; h: hour; CAM: chorioamnionitis; PPH: postpartum hemorrhage; NICU: neonatal intensive care unit PPH was defined as intrapartum blood loss ≥500 g in vaginal deliveries or ≥1,000 g in cesarean deliveries. A low Apgar score was defined as a 5-minute Apgar score <7. The chi-square test was used to compare categorical variables, while the Wilcoxon rank-sum test was applied to compare continuous variables.

	Oxytocin Group N = 60	DVI Group N = 60	Test Statistics	P-value
Maternal outcomes				
Vaginal delivery, n (%)	44 (73.3)	47 (78.3)	ⅹ^2^=0.41	0.52
Time from PROM to IOL, h, median (IQR)	27.7 (23.9-32.5)	27.5 (20.6-33.2)	Z =1.05	0.29
Time from PROM to delivery (all cases), h, median (IQR)	51.9 (37.4-72.2)	47.0 (36.1-64.6)	Z =1.07	0.28
Time from PROM to delivery (only vaginal delivery), h, median (IQR)	50.5 (36.0-68.3)	46.4 (33.9-52.6)	Z =1.14	0.25
Use of oxytocin, n (%)	60 (100)	28 (46.7)	ⅹ^2^=43.64	<0.0001
Clinical CAM, n (%)	8 (13.3)	5 (8.3)	ⅹ^2^=0.78	0.56
Intrapartum blood loss (g), median (IQR)	480 (273-758)	421 (272-634)	Z=0.13	0.26
PPH, n (%)	21 (35.0)	18 (30.0)	ⅹ^2^=0.34	0.56
Neonatal outcomes				
Birth weight, g median (IQR)	2962 (2684-3238)	2987 (2669-3119)	Z=0.54	0.59
Gestational age, weeks median (IQR)	39.5 (38.6-40.5)	39.6 (38.6-40.3)	Z=0.03	0.97
Low Apgar score, n (%)	0	0	-	-
NICU admission, n (%)	2 (3.3)	0	ⅹ^2^=2.03	0.5
Neonatal mortality, n (%)	0	0	-	-

Subgroup analysis results limited to cases of vaginal delivery are shown in Table 3. No differences were observed in maternal background characteristics. The rates of vaginal delivery within 24 and 48 hours from the start of labor induction were 26 (59.1%) and 36 (81.8%), respectively, in the oxytocin group and 31 (66.0%) and 41 (87.2%) in the DVI group. Although not statistically significant, both rates were higher in the DVI group. Notably, only 17 (36.2%) of patients in the DVI group required oxytocin administration.

**Table 3:** Subgroup analysis of maternal and neonatal outcomes in vaginal delivery cases DVI: dinoprostone vaginal insert; IQR: interquartile range; BMI: body mass index; PROM: prelabor rupture of membranes; IOL: induction of labor; CAM: chorioamnionitis; PPH: postpartum hemorrhage; NICU: neonatal intensive care unit PPH was defined as intrapartum blood loss ≥500 g in vaginal deliveries or ≥1,000 g in cesarean deliveries. A low Apgar score was defined as a 5-minute Apgar score <7. The chi-square test was used to compare categorical variables, while the Wilcoxon rank-sum test was applied to compare continuous variables.

Outcome	Oxytocin Group (N = 44)	DVI Group (N = 47)	Test Statistics	p-value
Maternal outcomes				
Age, years old, median (IQR)	32.0 (29.3-38.0)	32.0 (30.0-36.0)	Z=-0.35	0.73
Height, cm, median (IQR)	162 (156-165)	159 (156-163)	Z=0.86	0.39
Pre-pregnancy weight, kg, median (IQR)	52.0 (48.0-57.0)	53.0 (49.0-60.0)	Z=-0.85	0.39
Pre-pregnancy BMI, kg/m^2^	20.1 (18.8-22.1)	20.7 (19.7-23.1)	Z=-1.04	0.3
Weight at delivery, kg, median (IQR)	63.5 (57.5-69.6)	63.9 (58.9-69.6)	Z=0.03	0.97
BMI, kg/m^2^, median (IQR)	24.5 (22.6-27.9)	25.0 (23.5-27.0)	Z=-0.21	0.83
Nulliparous, n (%)	35 (88.5)	38 (80.8)	x^2^=0.02	0.88
Delivery within 24 hours of IOL, n (%)	26 (59.1)	31 (66.0)	x^2^=0.46	0.5
Delivery within 48 hours of IOL, n (%)	36 (81.8)	41 (87.2)	x^2^=0.51	0.47
Use of oxytocin, n (%)	44 (100)	17 (36.2)	x^2^=41.90	<0.0001
Clinical CAM, n (%)	2 (4.6)	1 (2.1)	x^2^=0.42	0.52
Intrapartum blood loss (g), median (IQR)	354 (247-548)	350 (214-545)	Z=0.16	0.87
PPH, n (%)	14 (31.8)	13 (27.7)	x^2^=0.19	0.66
Neonatal outcomes				
Birth weight, g), median (IQR)	2919 (2682-3132)	2964 (2654-3110)	Z=0.12	0.91
Gestational weeks, week), median (IQR)	39.1 (38.4-40.1)	39.3 (38.3-40.0)	Z=-0.26	0.79
low APS, n (%)	0	0	-	-
NICU admission, n (%)	2 (4.55)	0	x^2^=2.18	0.14
Neonatal mortality, n (%)	0	0	-	-

## Discussion

The introduction of DVI for labor induction in cases of PROM with an unfavorable cervix resulted in a slightly higher vaginal delivery rate in the DVI group compared to the oxytocin-only group; however, this difference was not statistically significant. Similarly, no significant differences were observed between the two groups in terms of maternal or neonatal outcomes. In contrast, the frequency of oxytocin use was significantly lower in the DVI group.

Although the vaginal delivery rate was marginally higher in the DVI group, the difference was not statistically significant when compared to the oxytocin-only group. Maternal and neonatal outcomes were also comparable. Previous studies have reported that the use of prostaglandin E₂ (PGE₂) prior to oxytocin induction in PROM cases increased the rate of vaginal delivery within 24 hours in a randomized controlled trial (RCT) [8]. Additionally, a retrospective study found that controlled-release DVI yielded favorable outcomes [10]. Conversely, other studies have supported the use of oxytocin alone, citing shorter labor durations [6, 7, 15]. Therefore, no clear consensus exists on the optimal induction method for PROM. In the present study, while the vaginal delivery rate was slightly higher in the DVI group, the difference did not reach statistical significance when compared to the oxytocin-only group. Additionally, the incidence of clinical chorioamnionitis did not increase in the DVI group, and the short-term neonatal outcomes remained comparable between groups. There is an ongoing debate regarding whether cervical ripening should be performed in cases of PROM when the cervix is unfavorable. One RCT reported an increased incidence of chorioamnionitis when balloon dilation was used for PROM [11], while another RCT found no difference [12]. A 2021 meta-analysis, however, reported a chorioamnionitis incidence of 15 of 154 (9.7%) with balloon use, which was 3.2 times higher (95% CI: 1.17-8.70) than the 5 of 174 (2.9%) observed without balloon use [4]. Based on these findings, mechanical methods of cervical ripening are generally not recommended for PROM.

In contrast, findings on the intravaginal administration of PG agents have been inconsistent. Some studies report no difference in intrauterine infection rates [1], while others suggest a potential increase [7], contributing to ongoing controversy. However, a meta-analysis reported that intravaginal administration of PGE₂ did not significantly increase the risk of intrauterine infection [11]. In the present study, the DVI group had a lower incidence of clinical chorioamnionitis, suggesting that DVI did not contribute to infection risk.

The frequency of oxytocin use was significantly lower in the DVI group. Although the controlled-release DVI used in this study is primarily intended for cervical ripening, vaginally administered PGE₂ also exerts uterotonic effects. While prior studies involving women without PROM have shown that intravaginal PGE₂ reduces oxytocin requirements compared to mechanical ripening methods [16, 17], a similar trend was observed in the current study: only 17 (36.2%) of patients in the DVI group who achieved vaginal delivery required oxytocin. These findings suggest that DVI may reduce the need for oxytocin during labor induction.

This study was a retrospective analysis conducted at a single institution. Due to the nature of our tertiary care facility, selection bias could not be entirely avoided. Additionally, some variables may have failed to reach statistical significance owing to the limited sample size.

## Conclusions

In this retrospective study conducted at a single Japanese tertiary care facility, the use of DVI instead of oxytocin alone for labor induction in cases of PROM with an unfavorable cervix did not alter the vaginal delivery rate. However, no increase in chorioamnionitis or adverse neonatal outcomes was observed. Moreover, the introduction of DVI significantly reduced the frequency of oxytocin use. Although the differences observed in this study were not statistically significant, the trends toward higher vaginal delivery rates and shorter delivery times in the DVI group suggest that DVI may offer clinical benefits. Further prospective studies with larger sample sizes are warranted to validate these findings.
